# Cathelicidin-like Peptide for Resistant *Acinetobacter baumannii* Control

**DOI:** 10.3390/antibiotics15010077

**Published:** 2026-01-12

**Authors:** Elizabete de Souza Cândido, Danieli Fernanda Buccini, Elizangela de Barros Miranda, Regina Meneses Gonçalves, Amanda Loren de Oliveira Brandão, Valentina Nieto-Marín, Ana Paula Ferreira Leal, Samilla Beatriz Rezende, Marlon Henrique Cardoso, Octavio Luiz Franco

**Affiliations:** 1 Centro de Análises Proteômicas e Bioquímicas, Programa de Pós-Graduação em Ciências Genômicas e Biotecnologia, Universidade Católica de Brasília, Brasília 71966-700, DF, Brazil; betty.souza@gmail.com (E.d.S.C.); marlonhenrique6@gmail.com (M.H.C.); 2 S-Inova Biotech, Programa de Pós-Graduação em Biotecnologia, Universidade Católica Dom Bosco, Campo Grande 79117-900, MS, Brazil; dfbuccini@gmail.com (D.F.B.); elihbarros.you@gmail.com (E.d.B.M.); re.menegon.2013@gmail.com (R.M.G.); amandalorend@gmail.com (A.L.d.O.B.); valentina.nieto@udea.edu.co (V.N.-M.); analeal.biotec@gmail.com (A.P.F.L.); samillab92@gmail.com (S.B.R.)

**Keywords:** antimicrobial peptide, biofilm, cutaneous infection, cathelicidin-like, skin

## Abstract

The growing global threat of antimicrobial resistance (AMR), particularly in cutaneous wound infections, represents a significant clinical and economic challenge. Biofilm formation by multidrug-resistant pathogens, such as *Acinetobacter baumannii*, often complicates healing and leads to therapeutic failure. Antimicrobial peptides (AMPs) are a promising alternative to conventional antibiotics due to their potent membrane-disrupting mechanism of action and lower propensity to induce resistance. **Background/Objectives**: This study aimed to evaluate the antibacterial, antibiofilm, and in vivo efficacy of four snake venom-derived cathelicidin-like peptides—Btn (15-34) and BotrAMP14 from *Bothrops atrox*, and Ctn (15-34) and CrotAMP14 from *Crotalus durissus*—against multidrug-resistant *A. baumannii*, *Escherichia coli*, and *Pseudomonas aeruginosa* clinical isolates from skin infections, with emphasis on *A. baumannii*, a WHO priority pathogen. **Methods**: Minimal Inhibitory Concentration (MIC), Minimal Bactericidal Concentration (MBC), and Minimal Biofilm Inhibitory Concentration (MBIC) were determined against *A. baumannii*, *Escherichia coli*, and *Pseudomonas aeruginosa*. Time-kill kinetics, hemolytic activity, and cytotoxicity assays were performed. A murine skin wound infection model was established to evaluate in vivo antibacterial efficacy and safety. **Results**: MIC/MBC values ranged from 0.78 to 25 µM against planktonic cells. In comparison, MBIC ranged from 1.56 to 12.5 µM against biofilms. BotrAMP14 eradicated *A. baumannii* within 4 min, while CrotAMP14 achieved bactericidal action in 20 min at 1.56 µM. Both peptides exhibited no hemolytic activity up to 128 µM and low cytotoxicity (IC_50_ > 128 µM). In vivo, BotrAMP14 and CrotAMP14 demonstrated significant antibacterial activity at 24 h and 48 h post-infection, respectively, surpassing that of meropenem. **Conclusions**: These findings suggest that BotrAMP14 and CrotAMP14 are promising topical antimicrobial agents for managing multidrug-resistant skin infections and may help address the urgent need for alternative therapies against antibiotic-resistant pathogens.

## 1. Introduction

Antimicrobial resistance (AMR) has become one of the most serious global public health threats, directly impacting the treatment of skin and soft-tissue infections (SSTIs). Infected and chronic wounds are frequently associated with multidrug-resistant (MDR) bacteria, such as *Acinetobacter baumannii*, *Pseudomonas aeruginosa*, and *Staphylococcus aureus*, which complicate therapy, delay healing, and increase healthcare costs [1,2]. According to the World Health Organization (WHO), *A. baumannii* is classified as a critical priority pathogen for which new antibiotics are urgently needed [3]. This scenario highlights the importance of developing new antimicrobial strategies to overcome resistance and biofilm-associated tolerance in skin infections.

The skin is the largest organ in the human body, and its primary function is to maintain physiological homeostasis and isolate and protect other organs and tissues from the external environment and pathogens [4]. When the skin is injured, whether by trauma, surgery, or disease, a process known as healing is activated. This healing process consists of four stages—hemostasis, inflammation, proliferation, and remodeling [5]. During these stages, the immune system recruits different cell populations to the wound site, which are responsible for signaling and executing the healing processes [6]. The combination of wound formation and changes in the individual’s immune response creates an ideal environment for bacterial growth and colonization. Necrotic tissue and various cellular debris found in the wound environment also provide nutrient-rich contact surfaces for bacterial attachment and subsequent biofilm formation [7], which can be problematic, as infection in open wounds hinders healing by dysregulating the immune response. In these situations, the presence of bacteria and biofilm formation may be associated with increased levels of pro-inflammatory cytokines and with degradation of the extracellular matrix and other proteins involved in wound healing [8,9]. When this occurs, and the acute wound does not heal properly, chronic or non-healing wounds can develop [10]. Chronic wounds affect the physical and emotional health of patients, and the treatment of these wounds is costly and lengthy, as it may require surgical intervention. The development of infection and the inability to heal can lead to amputation of limbs, resulting in disability. The emotional impact includes the development of depression and anxiety [10].

Currently, the management of infected wounds often relies on topical formulations or dressings loaded with antibiotics and antiseptic agents. However, the persistent and sometimes indiscriminate use of these conventional approaches has accelerated the selection of resistant strains, limiting treatment efficacy and motivating the search for alternative therapies [11].

The treatment of infected wounds involves the use of dressings, such as hydrogels, sponges, or films, loaded with various antibiotics, including gentamicin, ciprofloxacin, and tetracycline [12,13,14,15]. When highly contaminated, anti-infective agents, such as sodium hypochlorite [16], cyclic lipopeptides [17], silver-impregnated materials [18], and enzymatic debridement materials [19] can also be used. However, continued use of conventional therapies can lead to the development of resistance in pathogens to these treatments, especially to antibiotics. Given this challenge, the development of novel therapeutic molecules that can effectively target MDR pathogens while preventing resistance has become a research priority. The use of antimicrobial peptides (AMPs) has emerged as a promising option for treating infected wounds, due to their potent antimicrobial activity and lower likelihood of resistance compared to conventional antibiotics. Another advantage of using AMPs in wound care is that, in some cases, they can regulate the immune system, thereby aiding the patient’s healing process [20,21,22].

Among the diverse families of AMPs, cathelicidin-like peptides (Cathelicidin Antimicrobial Peptides—CAMPs) are particularly promising. Characterized by an a-helical amphipathic structure, these peptides can interact with and destabilize bacterial membranes with high efficacy and also possess immunomodulatory functions [23]. These specific peptides (Btn (15–34), Ctn (15–34), and their analogues BotrAMP14 and CrotAMP14) were selected because previous studies by our group demonstrated their potent antimicrobial activity, structural stability, and low cytotoxicity, making them ideal candidates for further investigation [23]. Their origin from snake venoms, a well-known source of bioactive molecules, further supports their potential as templates for new antimicrobial agents.

Thus, this work aimed to evaluate and compare the inhibitory and/or eradicatory effects of four cathelicidin-like peptides against resistant planktonic and biofilm cells of bacterial strains that mainly affect patients with cutaneous infections. Additionally, hemolysis, cytotoxicity, and a murine skin infection model were used to evaluate toxicity and antibacterial effects.

## 2. Results and Discussion

Peptides Btn (15–34) [24] and Ctn (15–34) [25], and their respective analogues BotrAMP14 (*Bothrops* cathelicidin analogue) [23] and CrotAMP14 (*Crotalus* cathelicidin analogue) [23] were synthesized by solid phase technique (F-moc) with a purity degree above 95%. For confirmation of peptide masses, we used MALDI-ToF analysis, showing *m/z* ions of 2477.57 Da, 1956.96 Da, 2370.44 Da, and 1820.25 Da, corresponding to Btn (15–34), BotrAMP14, Ctn (15–34), and CrotAMP14, respectively. Btn (15–34) [26] and Ctn (15–34) [25,27] had previously been reported as cathelicidins with strong potential to kill bacteria, including *Pseudomonas aeruginosa*, *Klebsiella pneumoniae*, and *Escherichia coli*.

The BotrAMP14 and CrotAMP14 peptides are rationally designed analogues derived from the parental fragments Btn (15–34) and Ctn (15–34), respectively. The complete amino acid sequences and structural characterization of Btn (15–34), Ctn (15–34), BotrAMP14, and CrotAMP14 are available in the original publications [23,26]. The design strategy, previously described by our group [23], aimed to shorten the original sequences (from 20 to 14 amino acids) while optimizing their physicochemical properties, including net positive charge and hydrophobic moment. These modifications were intended to enhance antimicrobial potency and reduce cytotoxicity. Therefore, the differences in antibacterial activity observed between the parental peptides and their analogues in the present study can be attributed to these sequence-driven alterations, which influence the α-helical amphipathic conformation and interaction with bacterial membranes [23].

The antimicrobial activity of the peptides was systematically evaluated against three multidrug-resistant Gram-negative clinical isolates, allowing a comparative assessment of their efficacy toward each bacterial species. Against *Acinetobacter baumannii* 003321216, all peptides demonstrated measurable inhibitory and bactericidal activity, although with distinct potency profiles. Btn (15–34) exhibited the lowest activity, with minimal inhibitory concentration (MIC) and minimal bactericidal concentration (MBC) values of 12.5 µM and 25 µM, respectively. Ctn (15–34) showed improved performance, inhibiting bacterial growth at 3.12 µM and displaying bactericidal activity at 6.25 µM. In contrast, both analogue peptides were markedly more potent. BotrAMP14 inhibited and eradicated the strain at 1.56 µM, while CrotAMP14 exhibited an identical inhibitory and bactericidal threshold of 1.56 µM, representing the highest efficacy observed for this species [26,28]. Focusing specifically on the antibacterial activity against *A. baumannii* 00332121, the peptides OH-CATH [27], Pt_CRAMP1 [25], crotalicidin (Ctn) [24,25,29], batroxicidin (BatxC) [24,25], and Hc-CATH [30] have been previously described as highly effective against clinical strains, which corroborates our results.

A comparable trend was observed for *Escherichia coli KpC^+^* 001812446. Btn (15–34) inhibited and eliminated the strain at 6.25 µM, whereas Ctn (15–34) displayed similar MIC and MBC values of 6.25 µM. Substantially enhanced activity was detected for the analogue peptides. BotrAMP14 achieved both inhibition and bactericidal action at 1.56 µM, demonstrating a fourfold improvement in potency compared to the parental fragments. CrotAMP14 also exhibited strong antibacterial activity, with MIC and MBC values of 3.12 µM, thus outperforming the original peptides and confirming its enhanced efficacy.

All peptides also displayed activity against *Pseudomonas aeruginosa* 003321199, though with clear differences in potency. Btn (15–34) inhibited and eradicated the strain at 6.25 µM. Ctn (15–34) showed superior activity with both MIC and MBC values of 3.12 µM. As observed for the other species, the analogue peptides exhibited the highest antimicrobial effectiveness. BotrAMP14 displayed exceptional potency, inhibiting and eliminating P. aeruginosa at concentrations below 0.78 µM, corresponding to a 4- to 16-fold improvement relative to its parental fragment. CrotAMP14 also demonstrated high activity, with MIC and MBC values of 1.56 µM.

Taken together (Table 1), these findings show that while all peptides possess relevant antibacterial properties, BotrAMP14 and CrotAMP14 consistently exhibit the greatest efficacy across the three multidrug-resistant Gram-negative pathogens evaluated. Their superior performance underscores the enhanced therapeutic potential of these analogues, particularly for combating clinically significant, drug-resistant bacterial infections.

As for the MBIC analyses (Table 1), Btn (15-34) exhibited antibiofilm activity against resistant strains of *A. baumannii* 00332121 and *E. coli KpC+* 001812446 at a concentration of 12.5 µM. Its analogue, BotrAMP14, was active, achieving total biofilm inhibition at 1.56 µM for both strains, corresponding to a concentration eight times lower than that of its parental fragment. In contrast, Ctn (15–34) inhibited biofilm formation with CIMBs of 12.5 and 6.25 µM for bacterial strains *A. baumannii* 00332121 and *E. coli KpC+* 001812446, respectively (Table 1). CrotAMP14 showed better efficacy in antibiofilm action against both strains, inhibiting *A. baumannii* 00332121 at 3.12 µM and *E. coli KpC+* 001812446 at 1.56 µM, demonstrating concentrations 4 times lower compared to the parental peptide fragment.

**Figure 1:**
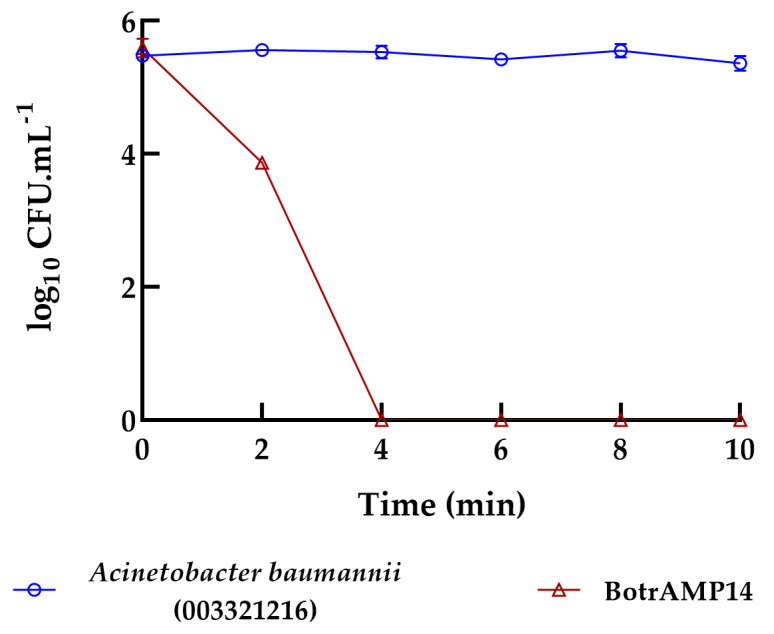
Time-kill kinetics of BotrAMP14 peptide towards *Acinetobacter baumannii* ATCC 003321216 strain. BotrAMP14 peptide was evaluated for 10 min, and the experiment was measured every 2 min. Three independent experiments were performed.

**Figure 2:**
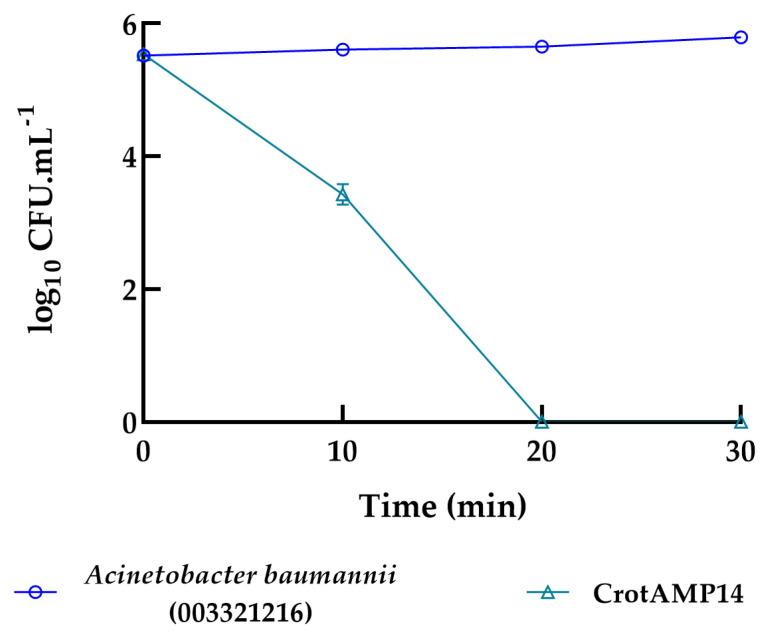
Time-kill kinetics of CrotAMP14 peptide towards *Acinetobacter baumannii* ATCC 003321216 strain. CrotAMP14 experiment was measured every 10 min for 30 min. Three independent experiments were performed.

BotrAMP14 (Figure 1) and CrotAMP14 (Figure 2) were evaluated using time-kill kinetics (TKK) assays due to their high activity. Bacterial growth control was confirmed in Muller-Hinton broth in the absence of the peptide, with a logarithmic concentration of 1 × 10^6^ CFU.mL^−1^. Both peptides exhibited concentration-dependent bactericidal activity against clinical isolate of *A. baumannii* 00332121. Remarkably, within 4 min of exposure, the BotrAMP14 completely eradicated the bacteria at 1.56 µM, equivalent to the MBC, and remained constant throughout the experiment (10 min) (Figure 1).

At its bactericidal concentration (1.56 µM), the CrotAMP14 peptide eradicated the bacterial strain *A. baumannii* 00332121 after 20 min of exposure. Although CrotAMP14 takes longer to initiate its bactericidal action, total cell reduction was observed after 20 min of exposure, remaining stable over the 30 min evaluation period (Figure 2). In a previous study by our research group regarding the characterization of these peptides, both BotrAMP14 (8.1 µM) and CrotAMP14 (2.2 µM) caused *E. coli* membrane damage from 5 to 60 min, as evaluated by scanning electron microscopy [23]. Interestingly, in that study, the differences in the peptides’ concentration did not directly influence on the killing time through pore formation on the bacterial membrane. Nonetheless, in the present study, by using the same peptides’ concentration in the time-kill kinetics assay, we observe a difference at the eradication time. Bearing this in mind, we must consider that here the assays were performed with an *A. baumannii* clinical isolate strain, whereas in our previous study we used an *E. coli* ATCC. These two species may highly diverge not only in metabolic terms, but also in the lipopolysaccharide chemotypes exposed on the Gram-negative outer membranes [28,29,31,32,33], which is the main target of BotrAMP14 and CrotAMP14. Additionally, our previous study shed some light over the structural scaffolds of these two peptides, highlighting how the structural flexibility/rigidity would influence on their antibacterial effects as a function of time (SEM analysis). These structural fluctuations [34], characteristic of short peptides such as BotrAMP14 and CrotAMP14, may be highly correlated with the results obtained in the time-kill assays and the different eradication times.

Studies exploring the components of snake venoms, whether from the Viperidade or Elapidae family, show that their antibacterial potential and their hemolytic and cytotoxic activities are all linked to the portion of molecules of protein origin. This obliges us to carry out a meticulous and precise analysis of the potential of our bioinspired peptides. In vitro biological activity of Btn (15–34), BotrAMP14, Ctn (15–34), and CrotAMP14 was evaluated against mouse erythrocytes and Raw264.7 cells for hemolytic and cytotoxic potential using the MTT assay. BotrAMP14 induced concentration-dependent hemolysis, displaying an estimated IC_50_ of approximately 92 µM. In contrast, Btn (15–34), CrotAMP14 and Ctn (15–34) did not reach 50% hemolysis at the highest tested concentration (128 µM), and their IC_50_ values were therefore considered above the tested range (Table 1 and Supplementary Figure S1). Furthermore, Btn (15–34), BotrAMP14, Ctn (15–34), and CrotAMP14 did not exhibit cytotoxic activity against Raw 264.7 cells until the highest concentration tested, 128 µM. BotrAMP14 and CrotAMP14 presented the best results and were selected for in vivo tests.

**Figure 3:**
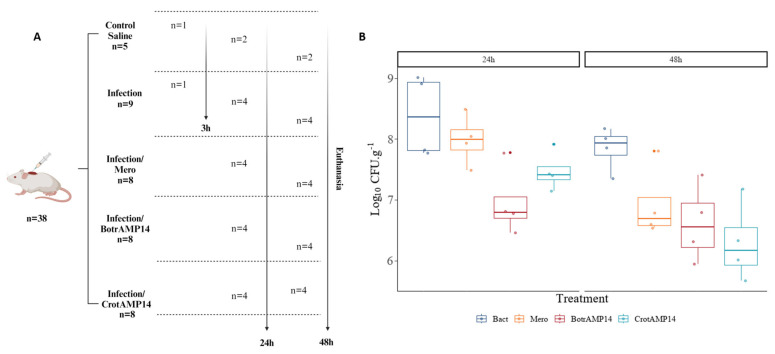
In vivo evaluation of the antibacterial activity of BotrAMP14 and CrotAMP14 in a murine skin wound infection model caused by multidrug-resistant *A. baumannii* 00332121. (**A**) Experimental design. (**B**) Bacterial load (log_10_ CFU.g^−1^ of tissue) in infected skin after 24 h and 48 h of treatment. Animals received topical applications of BotrAMP14 or CrotAMP14 (16 µM) or meropenem (Mero) (48 µM, antibiotic control) at 2, 12, 24, and 36 h post-infection. The bacterial control group consisted of infected animals that received no treatment. Data are expressed as median ± interquartile range. Statistical comparisons were performed using one-way ANOVA followed by Tukey’s post hoc test (*p* < 0.05 compared with bacterial control). Image created with BioRender.com.

To evaluate the antibacterial effect of BotrAMP14 and CrotAMP14 against *A. baumannii* 00332121 in a skin wound mouse model, the bacterial skin burden after the treatments was compared. A peptide concentration of 16 µM was selected for the in vivo assays based on its lack of cytotoxicity and hemolytic activity observed in vitro (Table 1) and to ensure a safe therapeutic margin. The infection control group included 9 animals (*n* = 9), the saline control group 5 animals (*n* = 5), and the meropenem and peptide treatment groups 8 animals each (*n* = 8), in accordance with the 3Rs principle (Replacement, Reduction, and Refinement). The animals received multiple topical treatments (at 2, 12, 24, and 36 h post-infection), and bacterial load was quantified at 24 h and 48 h post-infection (Figure 3). Statistical tests were applied to the raw data to compare the peptide’s effects at 24 and 48 h post-infection on the CFU recovery per gram of skin tissue (Log10 CFU.g^−1^). For this, the effect of each treatment was independently compared with the bacterial control, in which animals were wounded and infected but did not receive any treatment after infection. One animal from the bacterial control group was euthanized 3 h after infection and the excised skin tissue was plated to confirm the bacterial burden.

As shown in Figure 3, a statistically significant difference was observed in log CFU recovery between the treated and bacterial control groups at 24 h. However, after 48 h of treatment, the peptide did not exhibit a statistically significant difference in antibacterial effect compared with the 48 h bacterial control treatment group. This outcome likely reflects a temporal difference in antibacterial dynamics rather than loss of efficacy. BotrAMP14 produced an early and pronounced reduction in bacterial load at 24 h. In contrast, the effect was not sustained at 48 h—possibly due to bacterial regrowth or reduced peptide availability after the final application at 36 h. In contrast, CrotAMP14 exhibited a delayed but significant antibacterial effect at 48 h, while no significant difference was observed at 24 h, suggesting distinct pharmacodynamic profiles between the two peptides. It is essential to note that even when the antibiotic concentration was 3 times higher than the peptide concentration, no differences were observed at any of the evaluated times compared to the infection control treatment.

The results of the in vivo assay demonstrated the efficacy of both peptides in controlling infection in mouse skin wounds caused by *A. baumannii* 00332121. These results also showed a difference in the time of action between the two peptides. This could be related to a possible difference in the type of affinity interactions between the peptides and the bacterial membrane, and thus their mechanism of action. This has already been observed by other authors, who have noted that the same concentrations of different peptides cause bacterial death at varying rates, either in a linear or non-linear manner [35].

Although synergistic interactions between antimicrobial peptides and antibiotics were not investigated in this study, the present results highlight the distinct antibacterial behavior of BotrAMP14 and CrotAMP14 compared to meropenem, underscoring their therapeutic potential against multidrug-resistant *A. baumannii* 00332121. Considering their membrane-disruptive mechanism of action [23], these peptides could, in principle, facilitate antibiotic entry and complement the activity of intracellularly acting drugs. This complementary mode of action has been described as a possible basis for synergy between AMPs and antibiotics in previous studies [36,37,38]. While this was beyond the scope of the current work, the possibility of combining BotrAMP14 or CrotAMP14 with conventional antibiotics may represent an interesting strategy for future translational studies aimed at improving therapeutic outcomes in MDR wound infections.

This study demonstrates that the snake venom-derived cathelicidin-like peptides BotrAMP14 and CrotAMP14 possess strong therapeutic potential against multidrug-resistant pathogens associated with skin infections, particularly *Acinetobacter baumannii*. Both peptides exhibited potent antibacterial and antibiofilm activity, rapid bactericidal kinetics, and negligible hemolytic and cytotoxic effects, outperforming their parental fragments and, in key parameters, the clinically used antibiotic meropenem. Their efficacy in a murine wound infection model further supports their suitability as candidates for topical antimicrobial applications. Despite these promising findings, important challenges remain for future development. The distinct temporal patterns of in vivo activity highlight the need for deeper investigation into pharmacodynamics, peptide–membrane interactions, and local tissue stability. Moreover, optimization of formulation, delivery strategies, and long-term safety must be addressed to ensure translational viability. Overall, this work establishes BotrAMP14 and CrotAMP14 as compelling lead molecules for next-generation antimicrobial development. Their broad-spectrum activity, low toxicity, and in vivo efficacy provide a strong foundation for advancing cathelicidin-inspired peptides toward clinical applications aimed at combating drug-resistant skin pathogens.

## 3. Materials and Methods

### 3.1. Peptide Synthesis

Peptides Btn (15-34) fragment of batroxicin and its analog BotrAMP14, Ctn (15-34) from crotalicidin and its analog CrotAMP14 were synthesized by AminoTech Research & Development Ltda. (São Paulo, Brazil) using 9-fluorenylmethylenoxy-carbonyl (F-moc) solid-phase chemical synthesis followed by high-performance liquid chromatography (HPLC), with purification to 95% purity. The mass and sequence of the synthesized peptides were confirmed by mass spectrometry, MALDI-ToF/ToF Ultraflex III (Bruker Daltonics, Billerica, MA, USA) using the matrix-assisted laser desorption ionization-time of flight (MALDI-ToF) technique.

### 3.2. Microorganisms

Clinical bacterial strains of *A. baumannii* (003321216), *E. coli* KpC+ (001812446), and *P. aeruginosa* (003321199) were used in this research. Strains were kindly donated by Laboratório Central de Saúde Pública—LACEN of Brasília, Brazil.

### 3.3. Minimal Inhibitory Concentration (MIC) Assays

MIC assays were performed as described by Wiegand et al. [39]. Initially, bacteria were seeded on Muller-Hinton agar (MHA) plates and incubated at 37 °C for 18–24 h. Subsequently, isolated colonies of each bacterium were inoculated into tubes with 5 mL of Muller-Hinton broth (MHB; three biological replicates) and incubated at 37 °C under agitation at 200 rpm overnight. After this period, 1:50 dilutions of the overnight cultures were prepared in MHB and grown to half-logarithmic phase in triplicate. Then, MIC tests were performed in 96-well plates. Thus, the bacteria were exposed to concentrations between 0.78 and 25 μM of the evaluated peptides, with the final bacterial concentration in each well, being (2–5 × 10^6^ CFU.mL^−1^). Bacterial cultures in MHB were used as positive growth controls. All tests were performed with three biological replicates. Plates were incubated at 37 °C for 18 h and optical density readings were taken at 600 nm. The MIC was determined as the lowest concentration of peptides or antibiotic that inhibited 100% of bacterial growth. Bacterial growth inhibition was assessed by measuring the absorbance at 600 nm using a microplate reader (Bio-Tek Instruments, Winooski, VT, USA).

### 3.4. Minimal Bactericidal Concentration (MBC) Assays

Analysis of the antibacterial activity of Btn (15–34), BotrAMP14, Ctn (15–34), CrotAMP14, and the meropenem control was performed according to the Clinical and Laboratory Standards Institute (CLSI) [40]. Peptides were diluted two-fold in the range of 25 μM to 0.78 μM. Finally, the incubated treatments for the MIC determination were plated on MHA using the drop plate (DP) method. The MHA Petri dishes were incubated at 37 °C for 24 h, and the concentrations that inhibited bacterial growth were reported as the MBC.

### 3.5. Minimal Inhibitory Concentration of Biofilms (MBIC) Assays

For MBIC [41] determination, the peptides were microdiluted [39], and the lowest concentration that prevented at least 50% of biofilm formation was reported as the MBIC. In addition, the highest concentration of 25 μM was used as a treatment against the biofilms, and the biofilms were incubated at 37 °C for 24 h. The minimum biofilm eradication concentration (MBEC) is reported as the concentration that eradicates at least 50% of the pre-formed biofilm. Planktonic cell growth was measured at OD 600 nm and subsequently removed. 96-well round-bottom plates were washed twice with deionized water, and adherent cells were stained with 0.01% crystal violet for 20 min. The plates were washed twice again with deionized water and dried at room temperature. Subsequently, the adherent cells were solubilized with 110 μL of 70% ethanol. Biofilm formation was analyzed using a microplate reader at OD 595 nm.

### 3.6. Bacterial Killing Experiments

A killing experiment [42,43] was performed with 1:100 dilutions of overnight cultures of *A. baumannii* 00332121 in the absence or presence of BotrAMP14 and CrotAMP14 at 1.56 µM. The time-kill kinetics curves for BotrAMP14 and CrotAMP14 were evaluated over 10 min and 30 min, respectively. After each treatment, 10-fold serial dilutions were performed; bacteria were plated on Muller-Hinton Agar and allowed to grow overnight at 37 °C, after which colony-forming unit (CFU) counts were recorded. Three independent experiments were performed.

### 3.7. Cytotoxicity Assay

The cytotoxicity of Btn (15-34), BotrAMP14, Ctn (15-34) and CrotAMP14 was tested by 3-(4,5-dimethylthiazolyl-2)-2,5-diphenyltetrazolium bromide (MTT) assay [44,45]. The RAW 264.7 murine macrophage cell line (BCRJ 0212) was maintained under sterile conditions in Dulbecco’s Modified Eagle Medium (DMEM; Sigma, St. Louis, MO, USA) supplemented with 10% fetal bovine serum (FBS; Sigma) at 37 °C in a humidified atmosphere containing 5% CO_2._ The cell line was acquired from Banco de Células do Rio de Janeiro (BCRJ 0212 https://bcrj.org.br/celula/raw-2647/ (accessed on 6 January 2026)). Cells were cultured in 75 cm^2^ flasks (Kasvi, Curitiba, Brazil) until reaching approximately 90% confluence, detached, and centrifuged at 1500 rpm for 5 min at room temperature. Peptide concentrations ranging from 3.9 μM to 128 μM were added to 96-well plates containing 2 × 10^5^ Raw 264.7 cells per well and incubated at 37 °C for 24 h. Cell numbers were measured at 540 nm using a microplate reader (Thermo Scientific Multiskan Britain, Abingdon, England). Percentages of cell viability were calculated in relation to the untreated cell control.

### 3.8. Hemolytic Assay

Mouse red blood cells were used to measure the hemolytic effect of all peptides from 3.9 to 128 μM. 1% (*v*/*v*) Triton X-100 and red blood cells (RBCs) in PBS were used as positive and negative controls, respectively. Sample absorbance was measured at 415 nm using a microplate reader (Thermo Scientific Multiskan Britain, England). Experiments were performed in triplicate. Hemolysis values were normalized to a positive control (0.1% Triton X-100), which was considered as 100% hemolysis, and to phosphate-buffered saline (PBS) as the negative control (0% hemolysis). The IC_50_ values were determined by fitting the normalized data to a four-parameter logistic (4PL) nonlinear regression model. When 50% hemolysis was not achieved within the tested concentration range, IC_50_ values were considered not reached and reported as greater than the highest tested concentration.

### 3.9. Skin Wound Infection Mouse Model

To evaluate the in vivo antibacterial potential of BotrAMP14 and CrotAMP14, a skin wound mouse model was used [46]. Five groups of eight-week-old Swiss mice, all infected with *A. baumannii* 00332121, were established. Two groups served as controls: one untreated and one treated with saline. The other three groups were treated with Meropenem (Mero), BotrAMP14, and CrotAMP14. Mice were anesthetized with ketamine (150 mg.kg^−1^) and xylazine (7.5 mg.kg^−1^), and their backs were shaved. A linear, superficial skin wound was made, and five min later, the animals were inoculated with 1 × 10^8^ CFU.20 μL^−1^ of *A. baumannii* 00332121 diluted in PBS. After infection, the animals were treated at 2, 12, 24, and 36 h with BotrAMP14 or CrotAMP14, which were administered to the infected area at a final concentration of 16 μM. Animals were euthanized at 3, 24, and 48 h after infection, and the wounded skin area was excised and homogenized in PBS using surgical scissors. Finally, to quantify bacterial concentrations, the PBS samples were diluted and plated on MHA. All the procedures were approved by the Animal Experimentation Ethics Committee (CEUA) of the Universidade Católica Dom Bosco (UCDB), protocol number 024/2018. Data distribution was determined by Kurtosis and Shapiro–Wilk test. Differences between treatments and controls were evaluated by ANOVA-test with the post hoc Tukey’s HSD test. Statistical significance was set a priori as *p* < 0.05.

## 4. Conclusions

In this study, we demonstrated the potent antimicrobial activity of two cathelicidin-like peptides, BotrAMP14 and CrotAMP14, derived from snake venom, against multidrug-resistant strains associated with skin infections, particularly *A. baumannii* 00332121. Both peptides showed remarkable efficacy in inhibiting planktonic cells and biofilms in vitro, while also exhibiting low cytotoxicity and hemolytic activity. In vivo assays further confirmed their ability to reduce bacterial load in infected skin wounds, supporting their potential application in topical antimicrobial therapy.

These findings reinforce the therapeutic promise of bioinspired peptides as alternative strategies to combat antimicrobial resistance. The observed differences in the kinetics of bacterial eradication between the two peptides suggest distinct mechanisms of interaction with bacterial membranes, which merit further investigation. Future studies should focus on elucidating their molecular targets, improving peptide stability, and evaluating their pharmacokinetics and long-term safety in preclinical models. Overall, BotrAMP14 and CrotAMP14 represent valuable candidates for the development of next-generation antimicrobials targeting drug-resistant skin pathogens.

Importantly, this study represents the first translational evaluation of the snake venom-derived cathelicidin analogues BotrAMP14 and CrotAMP14. Unlike our previous report focused on peptide design and structural characterization [23], the present work demonstrates, for the first time, their efficacy against multidrug-resistant *A. baumannii* 00332121, including antibiofilm activity, rapid bactericidal kinetics, and in vivo therapeutic potential in a murine wound infection model. These results establish proof of concept for the clinical development of cathelicidin-inspired peptides as topical agents to treat MDR skin infections.

## Figures and Tables

**Table 1:** Biological activities from cathelicidin-like peptides Btn (15–34) and its fragment BotrAMP14, and Ctn (15–34) and its fragment CrotAMP14.

Antibacterial Activity				
Microorganism	MIC (MBC) [µM]			
	Btn (15–34)	BotrAMP14	Ctn (15–34)	CrotAMP14
*A. baumannii* 003321216	12.5 (25)	1.56 (1.56)	3.12 (6.25)	1.56 (1.56)
*E. coli* KpC+ 001812446	6.25 (6.25)	1.56 (1.56)	6.25 (6.25)	3.12 (3.12)
*P. aeruginosa* 003321199	6.25 (6.25)	0.78 (0.78)	3.12 (3.12)	1.56 (1.56)
**Antibiofilm Activity (MBIC)**				
*A. baumannii* 003321216	12.5	1.56	12.5	3.12
*E. coli* KpC+ 001812446	12.5	1.56	6.25	1.56
**Hemolytic activity [µM]**				
Murine erythrocytes	>128	128	>128	>128
**Cytotoxic activity (IC50)**				
Raw 264.7	>128	>128	>128	>128

IC50: Half Maximal Inhibitory Concentration; MBIC: Minimal Inhibitory Concentration of Biofilm; MIC: Minimal Inhibitory Concentration; MBC: Minimal Bactericidal Concentration.

## Data Availability

The original contributions presented in this study are included in the article/Supplementary Materials. Further inquiries can be directed to the corresponding author.

## Supplementary Materials

The following supporting information can be downloaded at: https://www.mdpi.com/article/10.3390/antibiotics15010077/s1, Supplementary Figure S1: Hemolysis percentage profile of antimicrobial peptides Btn(15–34) and BotrAMP14; and Ctn(15–34) and CrotAMP14. Hemolytic activity was evaluated using mouse erythrocytes. Hemolysis values were normalized to a positive control (0.1% Triton X-100), which was considered as 100% hemolysis, and to phosphate-buffered saline (PBS) as the negative control (0% hemolysis).

## Abbreviations

The following abbreviations are used in this manuscript:

AMP	Antimicrobial Peptides
Btn (15–34)	*Bothrops* cathelicidin 15
BotrAMP14	*Bothrops* cathelicidin analogue 14
Ctn (15–34)	*Crotalus* cathelicidin 15
CrotAMP14	*Crotalus* cathelicidin 14
MIC	Minimal Inhibitory Concentration
MICB	Minimal Inhibitory Concentration of Biofilm
MBC	Minimal Bactericidal Concentration
MBEC	Minimal Biofilm Eradication Concentration
CFU	Colonies Forming Units
IC50	Half Maximal Inhibitory Concentration
DP	drop plate
MTT	3-(4,5-dimethylthiazolyl-2)-2,5-diphenyltetrazolium bromide assay
F-moc	solid phase chemical peptide synthesis
HPLC	High Performance Liquid Chromatography
KpC+	*Klebsiella pneumoniae* carbapenemase positive
MHA	Muller-Hinton agar
MHB	Muller-Hinton broth
PBS	Phosphate-Buffered Saline
CEUA	Animal Experimentation Ethics Committee
UCDB	Universidade Católica Dom Bosco
