# Supplementary material for: Cathelicidin-like Peptide for Resistant Acinetobacter baumannii Control
Source: Antibiotics (Basel). 2026 Jan 12;15(1):77. doi: 10.3390/antibiotics15010077 (PMC12837634; doi:10.3390/antibiotics15010077)
Supplement: Supplementary file 1 [file antibiotics-15-00077-s001.zip › antibiotics-3952121-supplementary.pdf]

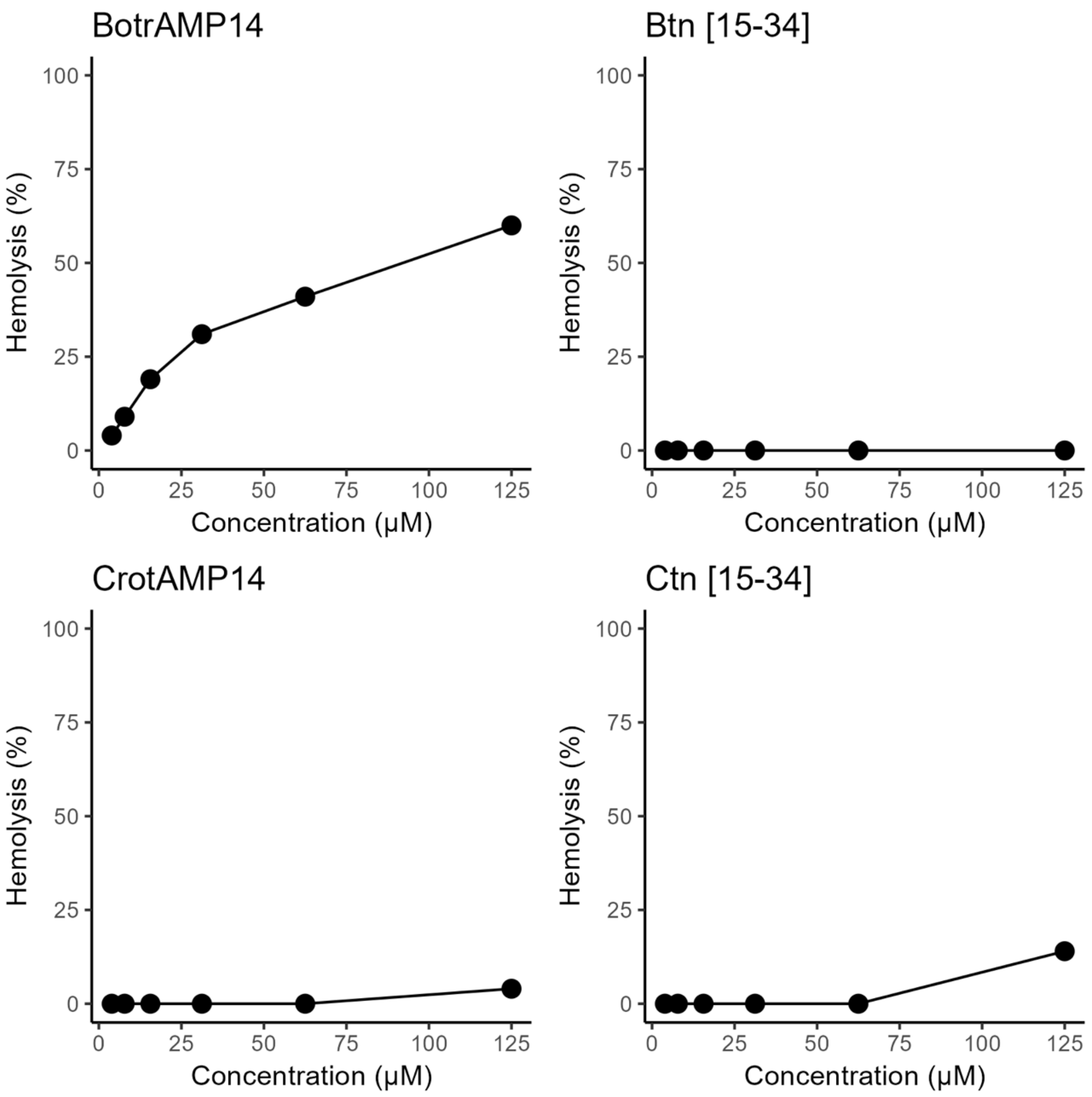

Supplementary Figure S1. Hemolysis percentage profile of antimicrobial peptides Btn(15-34) and BotrAMP14; and Ctn(15-34) and CrotAMP14. Hemolytic activity was evaluated using mouse erythrocytes. Hemolysis values were normalized to a positive control (0.1% Triton X-100), which was considered as 100% hemolysis, and to phosphate-buffered saline (PBS) as the negative control (0% hemolysis).
